# SANS and SAXS Investigation of the Melt State Structure in Disentangled Ultrahigh Molecular Weight Polyethylene

**DOI:** 10.1021/acsmacrolett.5c00100

**Published:** 2025-03-05

**Authors:** Aakash Sharma, Margarita Kruteva, Lutz Willner, Dario Romano, Lionel Porcar, Martin Dulle, Fuhai Zhou, Sanjay Rastogi, Dieter Richter

**Affiliations:** † 28334 Forschungszentrum Jülich GmbH, Jülich Centre for Neutron Science (JCNS-1: Neutron Scattering and Biological Matter), 52425 Jülich, Germany; ‡ 29616 CSIR-National Chemical Laboratory, Dr. Homi Bhabha Road, Pune, 411008 Maharashtra, India; § Division of Physical Sciences and Engineering, Department of Chemical Sciences, 127355 King Abdullah University of Science and Technology (KAUST), Thuwal 23955-6900, Kingdom of Saudi Arabia; ∥ 56053 Institut Laue-Langevin, B.P. 156, F-38042 Grenoble Cedex 9, France

## Abstract

Disentangled ultrahigh molecular weight polyethylene exhibits a time-dependent increase in rheology modulus when molten. This originates from its kinetically evolving heterogeneous microstructure consisting of disentangled and entangled regions. We report a quantitative analysis of this microstructure using X-rays and neutrons that capture the signatures of these regions. We analyze the absolute intensities to obtain the volume fraction and size distribution of the disentangled domains in the melt. Employing neutrons, we follow the changes in these parameters with time. The trends are qualitatively similar to those of the previous rheological observations. Our methodology also provides an experimental verification of the theoretical report by McLeish, T. C. B. *Soft Matter* **2007**, 3 (*1*), 83–87, which predicts the presence of high density disentangled domains in a low density entangled matrix. The analysis presented here is a useful instrument for unveiling the origin of differences in the properties of polymers obtained through different processing routes.

## Graphical abstract

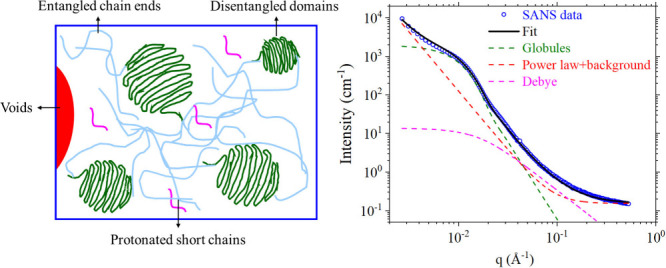

The utility of UHMWPE as an engineering plastic is well established in numerous applications like ballistic protection, sports, implants, automotive parts, etc.^1–6^ However, due to highly entangled polymer chains, conventional UHMWPE is characterized by enormous melt viscosity. This leaves the solution state processing as the most practical option, consuming ∼95% solvent,^7^ whereas the synthesis of disentangled ultrahigh molecular weight polyethylene (DUHMWPE) produces inherently disentangled single chain crystals.^8–10^ Melting of these crystals gives rise to a weakly entangled low viscosity melt that can be subjected to a sustainable solvent free process.^1,2,11–14^

Another point of interest arises from the unique physics of DUHMWPE. On heating it rapidly above its melting point (141 °C),^9^ a homogeneously entangled state is achieved.^9,15^ Whereas, if before melting DUHMWPE is subjected to annealing at a submelting temperature, a heterogeneous melt is obtained with coexisting entangled and disentangled zones. This transient state manifests itself as a time-dependent increase in the rheology plateau modulus.^8^ This hints at the nonequilibrium melt structure of the polymer, which could be a deciding factor for the transient properties.

The melting transitions of DUHMWPE have been an area of investigation for many years. Researchers have studied the melt structure and its evolution with time for DUHMWPE using various methodologies.^8–10,15–20^ Hawke et al. modeled the nonequilibrium melt state by assuming a larger diameter tube in the initial stage, which narrows in time.^16^ Vettorel and Kremer modeled the initially disentangled melt as nonoverlapping globules in simulations and showed that, for short chains, the entanglement process is unaffected by the topological constraints,^15^ whereas for long chains, these constraints have a major influence in generating the heterogeneous melt. Drakopoulos et al. were able to separately capture the relaxation of disentangled and entangled domains in the amorphous phase using rheology and dielectric spectroscopy.^18,19^ They further presented a model to estimate the activation energy associated with the entanglement of disentangled domains.

McLeish presented an elegant theory describing the melting process and melt structure in DUHMWPE.^17^ According to his theory, heating the single crystals of DUHMWPE at low temperatures results in the melting of chain ends. Whereas, the segments in the middle portion of the chain remain crystalline. This leads to the formation of entanglements at the chain ends. When the sample is heated to temperatures higher than the melting point, the middle portions also lose crystalline packing. However, due to the surrounding elastic mesh, these segments cannot expand to Gaussian coils. This results in the existence of a heterogeneous state with entangled chain ends of volume N^3/2^b^3^ and high density disentangled compartments with a volume same as that of crystals Nb^3^, where N is the degree of polymerization and b is the monomer size. With time, these compartments expand and entangle, giving rise to a slow increase in the shear modulus. Interestingly, this theory bases the unique behavior of DUHMWPE on the compartmentalization of melt with different densities in different zones. This density difference must result in an electron density difference, which could be utilized to experimentally observe these zones using scattering techniques.

To the best of our knowledge, there are no clear experiments in the literature that directly visualize the presence of disentangled domains in an entangled polymer. Here, we present a study of the structural evolution of DUHMWPE during melting. We report results from in situ small-angle X-ray scattering (SAXS) as well as small-angle neutron scattering (SANS) experiments.

We cocrystallize narrowly disperse hydrogenated chains of low molecular weight (MW = 4194 g/mol) with deuterated DUHMWPE using the synthesis protocol reported by Ronca et al.^21^ (details in Supporting Information, SI). SANS experiments were performed on the beamline D22 at Institut Laue-Langevin (ILL) in Grenoble (λ = 6 Å)^22^ using a home-built heating stage and SAXS experiments at Ganesha-Air from SAXSLAB/XENOCS equipped with a D2-MetalJet (Excillum) liquid-metal anode (λ = 1.314 Å) and PILATUS 300 K, Dectris detector. For SAXS, we employed a Linkam HFS600 heating stage under vacuum covered with Kapton film. The idea of cocrystallizing protonated chains with deuterated chains is to test the homogeneous mixing using neutrons, useful for future experiments. These differences are undetectable in X-rays. During experiments, we heat the sample rapidly (7 °C/min) to 130 °C and keep it at that temperature for 10 min. This was followed by slow heating (0.2 °C/min) to 150 °C and holding at that temperature for ∼2 h. During this, we perform scattering scans. After that the samples are heated to 165 °C and scanned continuously for 1 h. The heating protocol is the same for both SAXS and SANS. The intensities are corrected for instrumental and sample holder background and are converted to absolute units.

The temperature of the SANS heating stage was well calibrated, whereas Linkam HFS600 shows an offset of up to 3 °C at 160 °C without vacuum. This offset reduces further under a vacuum and at lower temperatures. The temperatures reported here are set temperatures. However, the findings remain unaffected by this small offset.

To confirm complete melting of crystals, we performed wide-angle X-ray diffraction (WAXD) at 150 and 165 °C and compared it to WAXD at 110 °C, as shown in SI, Figure S1. We noticed sharp crystalline peaks at 110 °C, whereas at both 150 and 165 °C, these peaks disappear and wide intensity distributions are observed that represent the amorphous phase. We know that, at 165 °C, the presence of any crystal is precluded.^9^ The similarity between WAXD from 150 and 165 °C conclusively proves the absence of crystals at 150 °C.

Next, we examine the nanoscopic structure development in DUHMWPE at 150 and 165 °C (Figure 1). At 165 °C, SAXS originates only from the background scattering (SI, Figure S2), which is created due to the retention of voids present in the as-synthesized polymer powder. A moderate compression of the sample in a vacuum minimizes these voids, but does not get rid of them completely. Application of extremely high compression was avoided to prevent the alteration of the inherent microstructure. In the SANS data at 165 °C (Figure 1), the void scattering is observed at low *q* as a power law along with the Debye scattering at intermediate *q* arising from the low MW protonated chains and background scattering (Bgr). The presence of the power law (*I* ∼ *q* ^3^) indicates that the sizes of these voids are larger than the accessible *q* range of SANS or SAXS. Nevertheless, their contribution to the net scattering could be easily removed by subtracting a power law and Bgr, as shown in Figure 1, leaving the scattering from Gaussian chains. Our prediction of this scattering (Debye form factor, eq 1) based on the volume fraction of the hydrogenated chains (ϕ_h_ = 5%), scattering length densities, and the radius of gyration (*R*_g_ = 89.8 Å) accurately matches with the experimental intensities. This confirms well-mixed protonated and deuterated chains.

$$I_{G}=\phi_{h}\left(1-\phi_{h}\right)V_{p}{\Delta \rho }_{h-d}^{2}\frac{2}{q^{4}R_{g}^{4}}\left[\exp\left(-q^{2}R_{g}^{2}\right)-1+q^{2}R_{g}^{2}\right]$$ 1

where *V*_p_ is the volume of a hydrogenated chain, Δρ_h–d_ is the scattering length density difference between hydrogenated and deuterated chains (6.88 × 10^10^ cm^–2^).

**Figure 1:**
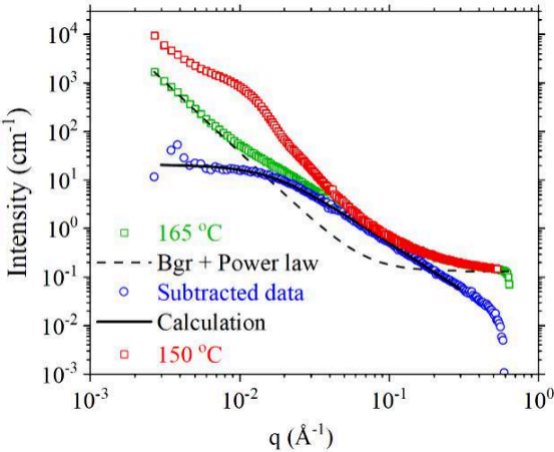
SANS data from DUHMWPE at 150 and 165 °C along with the background, power law subtraction, and calculated intensities for 165 °C data.

Figure 2 shows the SANS and SAXS data from samples at 150 °C. There is a power law at low *q*, similar to 165 °C, and background scattering at high *q*. Interestingly, we observe a prominent shoulder around *q* ∼ 0.01 Å^–1^, followed by a weak apparent minimum in both SAXS and SANS data.

**Figure 2:**
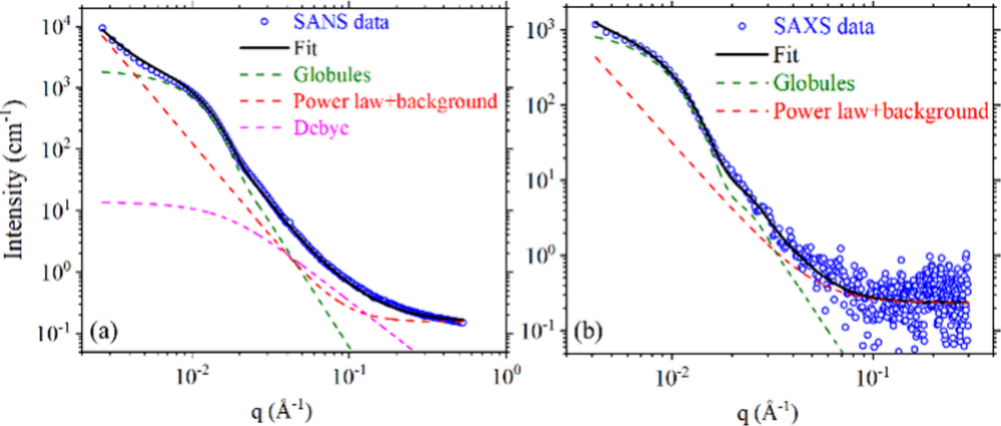
(a) SANS and (b) SAXS data from DUHMWPE samples measured at 150 °C, along with different contributions and the fits.

From WAXD data, we know that at 150 °C our sample is fully molten and hence amorphous. This omits the possible origin of the shoulder from crystal–crystal correlations. A comparison of intensities calculated from the known Debye function of protonated chains with the experimental results shows a substantial difference (Figure 2a). Therefore, this feature could be based on the presence of heterogeneous domains with separate entangled and disentangled chain segments.^17^ According to McLeish,^17^ above a critical MW, the elastic traps created by the entangled matrix overwhelm the confinement energy penalty borne by the disentangled domains trapped in an equivalent crystal volume. Therefore, the chains in the disentangled domains (*R* = N^1/3^b) are unable to expand to the size of Gaussian chains (*R*_g_ = N^1/2^b). This creates a net density difference between the disentangled and entangled parts. The scattering is based on the electron density difference or the scattering length density difference, both of which are affected by the mass density differences (Δρ_mass_). This allows us to visualize these disentangled domains in the SANS and SAXS data in the form of a shoulder and a weak minimum. The minimum indicates wide distribution in the size of disentangled domains, which could be due to the broad molecular weight distribution (MWD) of the as-synthesized polymer. For quantitative evaluations, we consider ρ_mass_ (disentangled globules) = ρ_mass_ (crystal)^23,24^ = 0.96 g/cm^3^, based on McLeish’s theory,^17^ whereas ρ_mass_ (entangled matrix) = 0.768 g/cm^3^.^25^

To visualize this scattering feature, the void content must be minimized by moderately compressing the sample under vacuum. Our attempts to visualize this scattering in the as-synthesized samples failed due to substantially high void scattering, which overwhelms all other contributions. The second important aspect is to anneal the samples at a temperature lower than the melting point of UHMWPE. This helps in the early entanglement of chain ends, as they have higher mobility compared with the rest of the chain. These entanglements act as a low-density elastic mesh. Having enough information on different domains in hand, we now describe the scattering intensities with minimal fit parameters.

The key to quantifying disentangled domains is the accurate calculation of theoretical absolute intensities and their comparison with the experimental values. Our attempts to fit data assuming a platelike form factor (based on the shape of crystalline lamellae), with the plate thickness fixed from literature, were not successful.^26^ Considering the isotropic stresses from the entangled matrix, we expect that the unexpanded disentangled domains attain the form of spherical globules. The scattering intensity *I* (eq 2) includes the globules, void scattering, Gaussian scattering, and a constant background:

$$I=\int_{0}^{\infty }{\Delta \rho }^{2}V{\left(\frac{3\sin qR-qR\cos qR}{{\left(qR\right)}^{3}}\right)}^{2}\phi P\left(R\right)dR+Aq^{-n}+I_{G}+C$$ 2

where Δρ is the electron density difference in case of SAXS (1.87 × 10^10^ cm^–2^) and scattering length difference in case of SANS (1.66 × 10^10^ cm^–2^) between globules and the entangled matrix (details in SI),^23–25^ *V* is the volume of a globule corresponding to its radius *R*, ϕ is the volume fraction of globules, *P*(*R*) is the function used to describe the radius distribution of globules, *A* is the prefactor to the power law for void scattering, *n* is the power (*n* ∼ 3; fixed based on 165 °C data), and *C* is the constant background. *I*_G_ is the SANS contribution from hydrogenated short chains (eq 1). *I*_G_ = 0 for the SAXS data.

To account for the heterogeneity in the globule size, we consider MWD of the as synthesized polymer, obtained by fitting the frequency sweep data.^8,27^ The MWD follows a log-normal shape. Based on this, we choose log-normal distribution for *P*(*R*). The fitting parameters are globule volume fraction (ϕ), mean (μ), and width (σ) of the distribution. Using Jscatter software,^28^ good quality fits were obtained for SANS and SAXS data as shown in Figure 2. To compare the fitted *P*(*R*) (SI, Figure S3) with the MWD of unmolten single crystals, we obtained the volumes of spherical globules from the fitted radii. The volume distribution is converted to corresponding MWD by multiplying with the crystal density (0.96 g/cm^3^). The corresponding MWD is shown in Figure 3.

**Figure 3:**
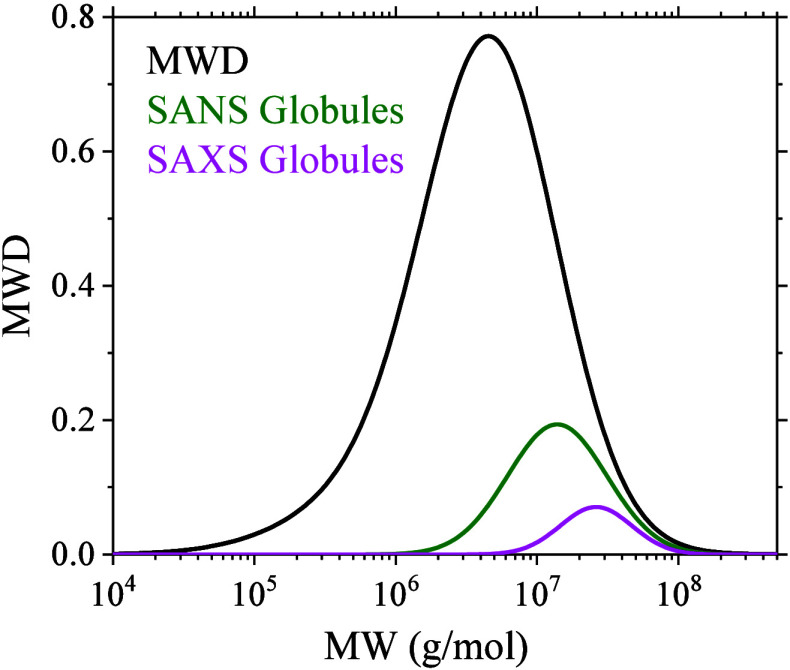
MWD of as-synthesized samples compared with the globule MWD obtained from SANS and SAXS fits at 150 °C.

Remarkably, the fitted *P*(*R*) and corresponding MWDs are contained within the crystal size distribution and are present toward the larger size range. This indicates complete melting of the short chains contributing to the entangled melt state along with the entangled ends of the long chains.

We obtain ϕ = 17% for SANS and ϕ = 4.47% for SAXS corresponding to the fits shown in Figure 2. The difference in the ϕ values from SANS and SAXS are due to manually applied compression, which varies from sample to sample. Following the described approach, we are not only able to visualize the disentangled domains, but also able to obtain quantitative numbers for corresponding ϕ and *P*(*R*). We note that these features are not visible at 165 °C, which should not be interpreted as the absence of disentangled domains. With an increase in temperature, the globules would expand, reducing Δρ_mass_ between the disentangled and entangled domains. Below a certain Δρ_mass_, the void scattering overwhelms the globule scattering. Lower values of ϕ and globule size can further contribute to this.

Having established the method for quantification of disentangled globules, we now follow the change in their size and ϕ with time. We kept the sample at 150 °C and for ∼2 h, performed continuous SANS scans at 1 scan/min. The measurements were repeated for two identical samples prepared in the same manner except the differences induced due to manual compression. The data was analyzed using the approach described above. The changes of ϕ, μ, and σ with time are presented in Figure 4.

**Figure 4:**
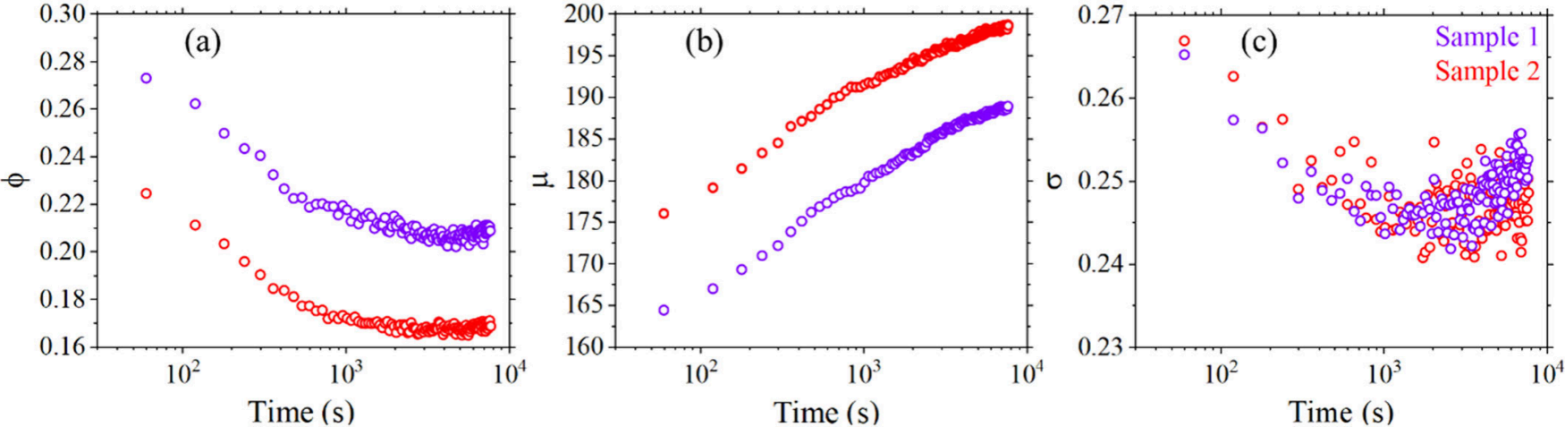
Evolution of (a) globule volume fraction, (b) mean radius, and (c) distribution width with time at 150 °C for two identical samples of DUHMWPE observed by SANS analysis.

ϕ continuously decreases with time and reaches an *apparent* slow decay after ∼2 × 10^3^ s. μ increases logarithmically, interpreted as expansion of globules as well as complete entanglement of short chains. This trend is qualitatively similar to the increase in rheology plateau modulus (*G*′) obtained at 160 °C.^8^ This important observation hints at the underlying relationship between the mechanical response of DUHMWPE and its melt structure. Although, our measurements are made at 150 °C, it would not be incorrect to expect a similar trend at 160 °C if there was zero void scattering. σ decreases logarithmically and reaches a plateau. An initial decrease in σ supports the entanglement of shorter chains in the beginning. Using these parameters, we plot the change in *P*(*R*) (SI, Figure S4a) and corresponding MWD (SI, Figure S4a) that shows a gradual decrease in heterogeneity with time.

For the MWD obtained at initial times (SI, Figure S4b, black curve), the lowest MW was ∼3 × 10^5^ g/mol. This is in remarkable agreement with the prediction of critical MW by Tom McLeish (∼10^5^ g/mol) above which heterogeneous melt should be observed.^17^ Critical MW was calculated by balancing the entanglement elastic free energy with the confinement entropy and accounting for the cooperative motion between chains. It should be noted that these calculations were performed for a monodisperse polymer, whereas, due to our broad MWD, an absolute quantitative comparison is not possible. Nevertheless, this preliminary agreement provides validation of the theory to some extent,^17^ which could in future be modified to include broad MWD.

A comparison of the DUHMWPE SAXS results with the entangled UHMWPE is in order for further validation. Therefore, we performed time dependent SAXS experiments on entangled UHMWPE synthesized through Ziegler–Natta route (SI, Figure S5). However, these samples exhibit no sign of heterogeneous melt (details in the SI). This establishes that scattering in Figures 1 and 2 correspond to disentangled globules and supports the applicability of our methodology for DUHMWPE.

The methodology described here serves as a robust tool to quantitatively understand the transient melt structure of DUHMWPE using scattering. This could be utilized to differentiate the characteristics of samples prepared with variations in the protocol. Presented study opens new pathways to understand the mechanical and thermal properties of DUHMWPE. Further, these results would help to build models that can relate the transient rheological response to the governing microstructural changes. Such a structure–property relationship is useful for optimization of processing. This requires a comprehensive study focused on different samples synthesized with variation in the MWD and different thermal history, which shall be part of future work.

## Supplementary Material



## Notes

(1) Synthesis protocols of protonated and deuterated polyethylene (2) Scattering length density calculations (3) Comparison of WAXD data at 110 °C, 150 and 165 °C (4) SAXS data at 165 °C (5) Comparison of radius distribution corresponding to as synthesized sample MWD with the fitted radius distribution of globules from SAXS and SANS (6) Evolution of globule radius distribution and corresponding MWD at 150 °C (7) SAXS and WAXD data for Ziegler–Natta UHMWPE (PDF)
