# Supplementary material for: SANS and SAXS Investigation of the Melt State Structure in Disentangled Ultrahigh Molecular Weight Polyethylene
Source: ACS Macro Lett. 2025 Mar 5;14(3):349–53. doi: 10.1021/acsmacrolett.5c00100 (PMC12794159; doi:10.1021/acsmacrolett.5c00100)
Supplement: Supplementary file 1 [file mz5c00100_si_001.pdf]

Supporting Information:

SANS and SAXS investigation of the melt state structure in disentangled ultra-high molecular weight polyethylene.

Aakash Sharma,*[a,b] Margarita Kruteva,*[a] Lutz Willner,[a] Dario Romano,[c] Lionel Porcar,[d] Martin Dulle,[a] Fuhai Zhou,[c] Sanjay Rastogi,*[c] and Dieter Richter[a]

[a]Forschungszentrum Jülich GmbH, Jülich Centre for Neutron Science (JCNS-1: Neutron Scattering and Biological Matter), 52425 Jülich, Germany.

[b]CSIR-National Chemical Laboratory, Dr. Homi Bhabha road, Pune, 411008, Maharashtra, India.

[c]Division of Physical Sciences and Engineering, Department of Chemical Sciences, King Abdullah University of Science and Technology (KAUST), Thuwal, 23955-6900 Kingdom of Saudi Arabia.

[d]Institut Laue-Langevin, B.P. 156, F-38042 Grenoble Cedex 9, France.

Corresponding Authors

*Margarita Kruteva: m.kruteva@fz-juelich.de
*Aakash Sharma: aakash.sharma@ncl.res.in
*Sanjay Rastogi: sanjay.rastogi@kaust.edu.sa

Synthesis of protonated chains:

The protonated polyethylene was synthesized by anionic polymerization of protonated-1,3-butadiene in benzene and subsequent saturation with hydrogen by means of a palladium on barium sulphate catalyst. The microstructure of the parent polybutadiene consists of 93% 1,4- (cis and trans) and 7% 1,2-addition of monomer units. Due to the 7% 1,2-content the final polyethylene carries on average 2 ethylene side branches per 100 -CH_2- groups. The polybutadiene was characterized by size exclusion chromatography in THF using a combination of refractive index/multi angle laser light detectors to determine the absolute number average molar mass, Mn = 40.44 kg/mol, and the polydispersity, Mw/Mn ≤ 1.01. The molar mass of the polyethylene, Mn=41.94 kg/mol, was calculated taking into account one H_2/butadiene repeat unit.

Synthesis of co-crystallized deuterated and protonated chains:

The polymerization was conducted in a Büchi 1.0 L jacketed stainless steel reactor vessel, equipped with a thermometer probe, catalyst/co-catalyst injection port, N_2/vacuum port, safety rupture disk, pressure transducer, monomer feeding port, solvent feeding port and a Büchi cyclone 75 stirrers equipped with a 4-bladed propeller. The reactor was left overnight under vacuum at 125 °C using a Huber Tango Fleur thermostat to reduce the impurities absorbed. Afterwards, at least three nitrogen/vacuum cycles were performed and the temperature was set to 10 °C. When the temperature reached equilibration, under constant stirring rate of 750 rpm, 250 ml of anhydrous toluene was transferred to the reactor vessel followed by the addition of 4 mL of a toluene solution of Methylaluminoxane, MAO (10 wt.%) to scavenge impurities. Separately, under nitrogen environment 1.25 g of protonated low molecular weight polyethylene (or protonated polybutadiene) were dissolved in 500 mL of anhydrous toluene at 40 °C in a round bottom flask (targeting 5 wt.% of protonated polyethylene in deuterated ultra high molecular weight polyethylene (DUHMWPE). Upon complete polymer dissolution, the solution was transferred to the reactor under nitrogen

environment. Absence of polymer precipitation when the polymer solution reached 10 °C was verified in a preliminary test. After temperature equilibration was reached, the nitrogen was vacuumed and 1.1 bar absolute pressure of deuterated-ethylene was set and maintained throughout the polymerization by means of a pressure regulator. After complete monomer solubilization and temperature equilibration, in a glovebox, 1 mL of a toluene solution of MAO (10 wt.%) and 1 mL of toluene were premixed for 5 minutes and used to dissolve and activate 5 mg of catalyst (bis[N-(3-tert-butylsalicydene)-2,3,4,5,6-pentafluoroanilinato] titanium (IV) dichloride complex). The polymerization reaction was initiated by the addition of the activated catalyst solution under deuterated ethylene flow. The polymerization was terminated after 60 minutes by venting the leftover monomer, followed by the addition of 10 ml of ethanol. The polymer was washed with ethanol and acetone in this order and subsequently dried at 40 °C in vacuum overnight. 0.7 - 1.0 wt.% (in respect to the polymer yield) of Irganox1010 (antioxidant) was dissolved in acetone and added to the polymer. The acetone was evaporated overnight until constant weight.

Molecular weight distribution for UHMWPE was obtained by fitting the frequency sweep data as elaborated in literature.[1,2] Corresponding, Mw = 7.975 x 10^6 g/mol and width of molecular weight distribution presented in the main manuscript (Figure 3, black curve), σ = 1.18.

Scattering length density calculations:

For UHMWPE at 150 °C, mass densities, $\rho_{crystal} \sim 0.96$ g/cc[3,4] and $\rho_{amorphous} = 0.768$ g/cc,[5] hence $\Delta\rho_{mass} = 0.192$ g/cc. Therefore, $\Delta\rho$ for SANS ($\Delta\rho_{SLD}$) and SAXS ($\Delta\rho_{e-}$) are calculated as:

$$\Delta\rho_{SLD} = \frac{SL\, N_A}{m}(\Delta\rho_{mass})\ and\ \Delta\rho_{e-} = \frac{n_e\, r_e\, N_A}{m}(\Delta\rho_{mass})$$

where, SL is scattering length of monomer, N_A is Avogadro's number, m is monomer mass, n_e is number of electrons per monomer and r_e is radius of electron.

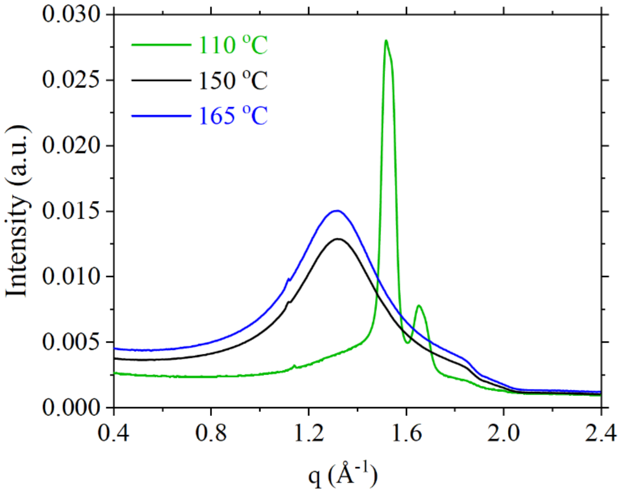

Figure. S1 Comparison of wide angle X ray diffraction data from DUHMWPE obtained at 110, 150 and 165 °C.

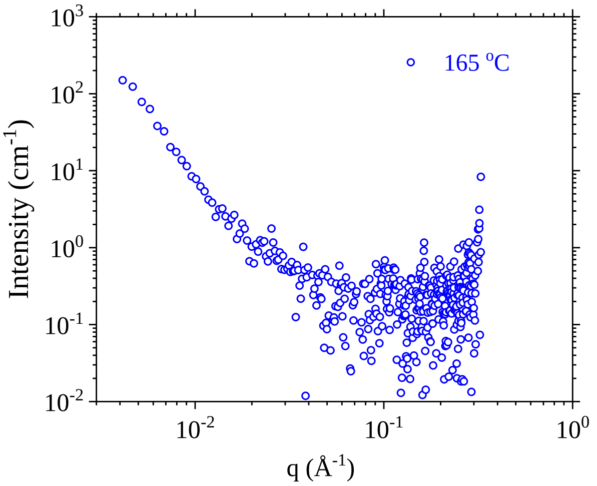

Figure. S2 Small angle X ray scattering data from DUHMWPE at 165 °C.

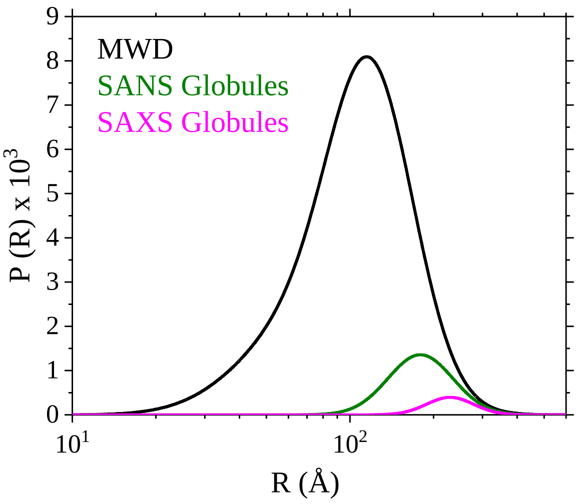

Figure. S3 Radius distribution obtained from the MWD of as synthesized samples compared with the globule radius distribution obtained from SANS and SAXS fits. To obtain radius distribution corresponding to the MWD of as synthesized samples, we calculate the volumes of crystals using the crystal density and MWD of polymer. This volume is converted to radius distribution of the corresponding sphere sizes.

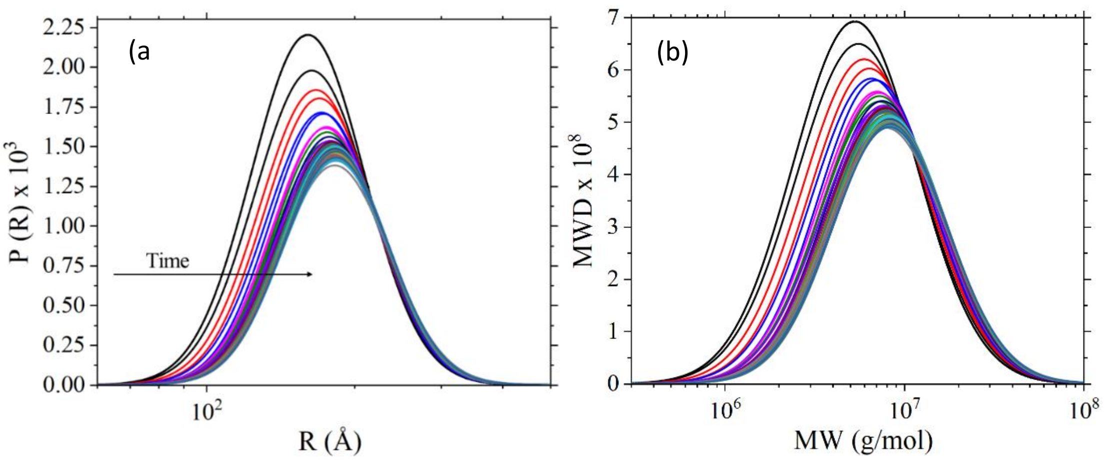

Figure. S4 (a) Evolution of globule radius distribution and (b) corresponding molecular weight distribution with time for sample kept at 150 °C.

SAXS and WAXD data for Ziegler Natta UHMWPE:

We investigated the conventional UHMWPE sample (M_w = 6.5 x 10^6 g/mol, PDI = 13.8) synthesized using the Ziegler Natta route in SAXS and WAXD experiments. Our aim was to search for a similar SAXS signal as shown by disentangled UHMWPE in the manuscript Figure 1 and 2. Differential scanning calorimetry shows that the Ziegler Natta UHMWPE sample has a melting temperature of 140.8 °C and is completely molten at 142 °C.

Following a similar protocol as reported in the manuscript, we heat this sample rapidly (10 °C/min) to 130 °C, followed by a slow heating (0.1 °C/min) to 150 °C. We then keep the temperature constant for ~4 hours while obtaining continuous scans. Figure S5 shows the SAXS (left) as well as WAXD (right) data on these samples obtained at 150 °C. Unlike the disentangled UHMWPE samples, the SAXS data only shows a continuous decay of intensity. We do not observe any shoulder as is observed for the disentangled samples.

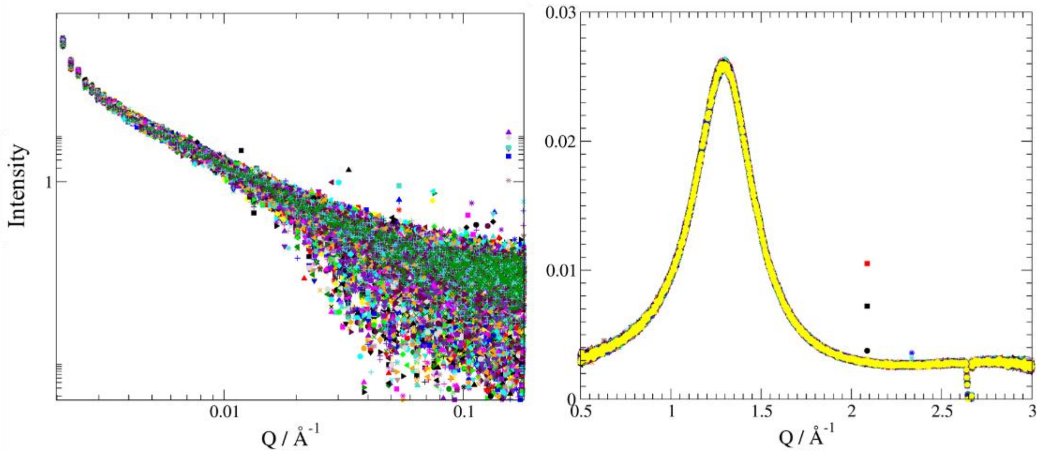

Figure. S5 (Left) Time dependent SAXS data at 150 °C and (Right) corresponding WAXD data for Ziegler Natta UHMWPE samples.

The comparison of disentangled UHMWPE samples from manuscript and the Ziegler Natta UHMWPE sample shows a clear difference in the SAXS intensities, while the WAXD data prove the absence of crystals in both UHMWPE. This proves that the SAXS signal in disentangled samples arises from the disentangled zones which are absent in the Ziegler Natta UHMWPE sample as we anticipated.

References:

(1) Pandey, A.; Champouret, Y.; Rastogi, S. Heterogeneity in the Distribution of Entanglement Density during Polymerization in Disentangled Ultrahigh Molecular Weight Polyethylene. *Macromolecules* **2011**, *44* (12), 4952–4960. https://doi.org/10.1021/ma2003689.

(2) Mead, D. W. Determination of Molecular Weight Distributions of Linear Flexible Polymers from Linear Viscoelastic Material Functions. *J. Rheol. (N. Y. N. Y).* **1994**, *38* (6), 1797–1827. https://doi.org/10.1122/1.550527.

(3) Kawai, T.; Keller, A. On the Density of Polyethylene Single Crystals. *Philos. Mag.* **1963**, *8* (91), 1203–1210. https://doi.org/10.1080/14786436308207345.

(4) Swan, P. R. Polyethylene Unit Cell Variations with Temperature. *J. Polym. Sci.* **1962**, *56* (164), 403–407. https://doi.org/10.1002/pol.1962.1205616410.

(5) Mark, J. E. *Physical Properties of Polymers Handbook*; New York, 2007. https://doi.org/10.1007/978-0-387-69002-5_25.
